# Diagnostic and prognostic features in adrenocortical carcinoma: a single institution case series and review of the literature

**DOI:** 10.1186/s12957-015-0527-4

**Published:** 2015-03-24

**Authors:** Kerollos N Wanis, Rani Kanthan

**Affiliations:** College of Medicine, University of Saskatchewan, Saskatoon, Canada; Department of Pathology and Laboratory Medicine, University of Saskatchewan, Saskatoon, Canada; Royal University Hospital, Room 2868G-Wing, 103 Hospital Drive, Saskatoon, Saskatchewan S7N 0W8 Canada

**Keywords:** Adrenocortical carcinoma, Sarcomatoid carcinoma, Immunohistochemistry, Reticulin, Diagnosis

## Abstract

**Background:**

Adrenocortical carcinoma is a rare cancer, with an incidence in the literature of 0.5 to 2 cases per million population per year. Adult adrenocortical carcinoma has a poor prognosis, underscoring the importance of identifying diagnostic and prognostic markers.

**Methods:**

We searched our laboratory database for all cases in the past 15 years with a diagnosis of adrenocortical carcinoma. The original slides were then reviewed for their histopathological features. A representative paraffin block was subjected to further immunohistochemical staining for Ki-67, inhibin, steroidogenic factor-1 (SF-1), p53, and Β-catenin. These slides were scored by the study pathologist who was blinded to all clinicopathological data. In addition, a comprehensive review of the relevant English literature in the past 15 years was conducted.

**Results:**

Eight cases were identified, including two adrenal sarcomatoid carcinomas. Seven of the eight cases had a disrupted reticulin network. Six of the eight tumors had >10% Ki-67 expression. Five of the eight tumors had >10% p53 expression. Positive inhibin immunohistochemical staining was seen in three of the eight tumors, and positive SF-1 staining was seen in five of the seven stained tumors. Abnormal Β-catenin intracellular accumulation was noted in four of the eight tumors. The two tumors in our series with sarcomatoid histology did not stain positively for SF-1 or inhibin.

**Conclusions:**

Eight cases of adrenocortical carcinoma, including two with sarcomatoid features are presented. The two sarcomatoid adrenocortical carcinomas in our series did not stain for SF-1 which suggests a possible *de novo* pathway of tumorigenesis for this rare variant. The reticulin staining method was a useful tool for rapid differentiation of adrenocortical adenomas and carcinomas. Diffuse p53 staining showed a trend for positive correlation with increased Ki-67 expression. Inhibin staining was inconsistently expressed in our cases of adrenocortical carcinoma. In conclusion, as adrenocortical carcinoma is a rare disease, we recommend future multicenter studies with appropriate sample sizes to further evaluate the efficacy of these diagnostic and prognostic markers.

## Background

Adrenocortical carcinoma (ACC) is rare and has a poor prognosis. Even for patients with resected tumors, the median survival is only 32 months in the United States [1]. While ACC is uncommon with an annual incidence of 0.5 to 2.0 per million people, adrenal incidentalomas are increasingly being recognized, due to the availability of superior imaging techniques, with a reported prevalence of 3% to 4% on abdominal CT scan [1-5]. Most adrenal incidentalomas are benign, while most malignant adrenal tumors are metastatic in origin [6]. As such, accurate diagnosis of malignant adrenocortical tumors, particularly distinguishing ACC from adrenal adenomas, is essential for management; however, accurate diagnosis continues to remain a challenge. There are continued significant areas of uncertainty regarding pathogenesis and risk assessment. In this context, several histopathological and immunohistochemical markers have emerged in the past two decades as additional adjuncts that include markers, such as steroidogenic factor-1 (SF-1) that may be useful in establishing the adrenocortical origin of an adrenal mass, while other markers, such as Ki-67 and p53, could help stratify tumors into prognostic groups.

The aim of this study is to discuss the diagnostic and prognostic features of ACC through a series of cases treated at our institution over a 15-year period in the context of a comprehensive relevant literature review. We compared immunohistochemical expression profiles of Ki-67, inhibin, SF-1, p53, and Β-catenin with histopathological features and patient outcome.

## Methods

### Pathological review

All patients in the Saskatoon Health Region Department of Pathology Laboratory Information System with a pathological diagnosis of adrenocortical carcinoma from 1998 to 2013 were identified. All cases were reviewed on a routine hematoxylin-eosin-stained slide according to the Weiss criteria to confirm the presence of ACC. Immunohistochemical studies using antibodies to Ki-67, B-catenin, SF-1, and p53 were performed on a representative deparaffinized tissue section by the avidin-biotin-peroxidase complex (ABC) technique after antigen retrieval using appropriate positive and negative controls in all cases. Negative controls were obtained by omission of the primary antibody from the staining procedure. The antibodies used with their sources, clones, antigen-retrieval techniques, and dilutions are listed in Table 1.

**Table 1:** **Antibodies used in this study**

**Antibody**	**Company and clone**	**Target retrieval solution (TRS)**	**Detection**	**Dilution**	**Link to spec sheet**
Beta catenin	Dako, B-catenin-1	High	Flex	1:200	Monoclonal mouse anti-human beta-catenin, clone β-catenin-1
Inhibin	Dako, R1	High	Flex	1:5	Monoclonal mouse anti-human inhibin α, clone R1
Ki67	Dako, MIB1	High	Flex	1:50	Monoclonal mouse anti-human Ki-67 antigen, clone MIB-1
Ki67 decal	Dako, MIB1	High	Flex	1:20	Same as above
p53	Dako, D0-7	High	Flex	1:100	Monoclonal mouse anti-human p53 protein, clone DO-7
SF-1	Invitrogen	Dako3in1 high pH on Dako PT instrument	Dako-envision + mouse	1:150	Monoclonal mouse [Mm] IgG1 anti-human clone N1665

IgG1, immunoglobulin G1; SF-1, steroidogenic factor-1.

Immunohistochemically stained slides were analyzed in the standard semi quantitative basis incorporating the intensity of the staining (mild, moderate, strong) coupled with the percentage of positively stained cells in a four-point scale: 0, no stain (up to 10% positive cells); 1, light (11% to 25% positive cells); 2, moderate (26% to 50% positive cells); 3, heavy (51% to 75% positive cells) and; 4, intense stain (76% to 100% positive cells). The cells were considered positive when more than 10% of them were stained with the respective antibodies. The reticulin framework of the tumors was examined by histochemical staining. The study pathologist was blinded to any clinical information prior to pathological review and interpretation for the purpose of this study.

The hospital charts for all patients were obtained and data was collected on age, sex, mode of presentation of the tumor, and available follow-up.

Upon application, this study was exempt from ethical approval by the University of Saskatchewan Biomedical Ethics Review Board.

### Literature review

A literature search using the National Library of Medicine Interface PubMed was conducted using the search terms ‘adrenocortical carcinoma’ and ‘adrenal cortical carcinoma’ which was limited to the English language from 1999 to present. The bibliographies of these manuscripts further identified relevant secondary sources.

A detailed review of the published large case series was undertaken. Large case series were defined as those having an ‘*n*’ greater than 300. These were compiled for tabulation.

## Results

### Pathological review

From an initial search list of 15 cases of possible ACC, eight patients with a ‘bonafide’ histopathological diagnosis of adrenocortical carcinoma were identified using the Weiss criteria as a guideline for diagnosis. There were five females and three males with an average age at diagnosis of 53.5 years (range 25 to 79).

Necrosis was the most common histopathological feature, found in six of eight tumors. Vascular invasion was identified in five of eight, marked nuclear pleomorphism in two of eight, and capsular invasion in one of eight. Increased mitotic rate greater than 5/50 high-powered fields (HPFs) was seen in seven cases. Atypical mitoses were easily observed in five cases. All cases had a diffuse architecture. Venous and sinusoidal invasion were present in five of the eight cases. In summary, seven cases had more than four Weiss criterion thereby meeting the histological criteria for ACC. In one case, though there was diffuse architecture with high grade nuclei and increased mitoses, diffuse necrosis, atypical mitoses, capsular, sinusoidal, or venous invasion were not identified. Reticulin staining revealed loss of the reticulin network in seven of the eight tumors. Ki-67 was overexpressed in all of the cases. Aberrant nuclear staining for p53 was noted in all but one case. Increased intracellular accumulation of Β-catenin was present in 50% of the cases. Three of the eight tumors had positive immunohistochemical staining for inhibin. Five of the eight tumors stained positively for SF-1, while two were negative, and one did not have adequate tissue available for staining. The results of immunohistochemical staining are summarized in Table 2.

**Table 2:** **Results of immunohistochemical staining for eight patients with adrenocortical carcinoma**

**Case number**	**Age**	**Sex**	**Ki-67 (%)**	**Β-catenin intracellular accumulation**	**P53 (%)**	**Inhibin**	**SF-1**
1	59	M	80	Present	85	Positive	Positive
2	25	F	40	Present	30	Negative	Positive
3	65	M	20	Absent	30	Negative	Negative
4	66	F	50	Absent	Focal	Positive	Tissue not available
5	79	M	10	Present	10	Positive	Positive
6	34	F	85	Present	90	Negative	Positive
7	68	F	60	Absent	60	Negative	Negative
8	32	F	10	Absent	Absent	Negative	Positive

F, female; M, male; SF-1, steroidogenic factor-1.

Two of the eight patients had tumors with sarcomatoid features. Interestingly, lack of SF-1, inhibin, and aberrant Β-catenin expression was noted in the sarcomatoid regions of these tumors.

### Clinical review

Seven of the eight patients had surgical resection. One patient presented with metastatic disease identified as ACC on lung biopsy. The median length of follow-up was 8 months (range 0.8 to 60.2). The most common presentation was an incidentally discovered mass during imaging (six of eight patients). One patient was presented with Cushing’s syndrome, and one was presented with symptoms related to metastatic lung disease. Three patients experienced recurrence, 2.3 to 6.5 months after resection. Of the four patients who died, three died with metastatic ACC and one died due to other medical comorbidities without ever having identified disease recurrence. The clinical data for all of the patients are summarized in Table 3.

**Table 3:** **Clinical data for eight patients with adrenocortical carcinoma**

**Case number**	**Age**	**Sex**	**Presentation**	**Time to recurrence (days)**	**Time to death (days)**
1	59	M	Cushingoid	N/A	N/A
2	25	F	Incidentally	119	N/A
3	65	M	Incidentally	N/A	N/A
4	66	F	Metastatic	0	25
5	79	M	Incidentally	N/A	353
6	34	F	Incidentally	195	254
7	68	F	Incidentally	68	223
8	32	F	Incidentally	N/A	N/A

F, female; M, male; N/A, not available.

### Sarcomatoid cases

**Figure 1:**
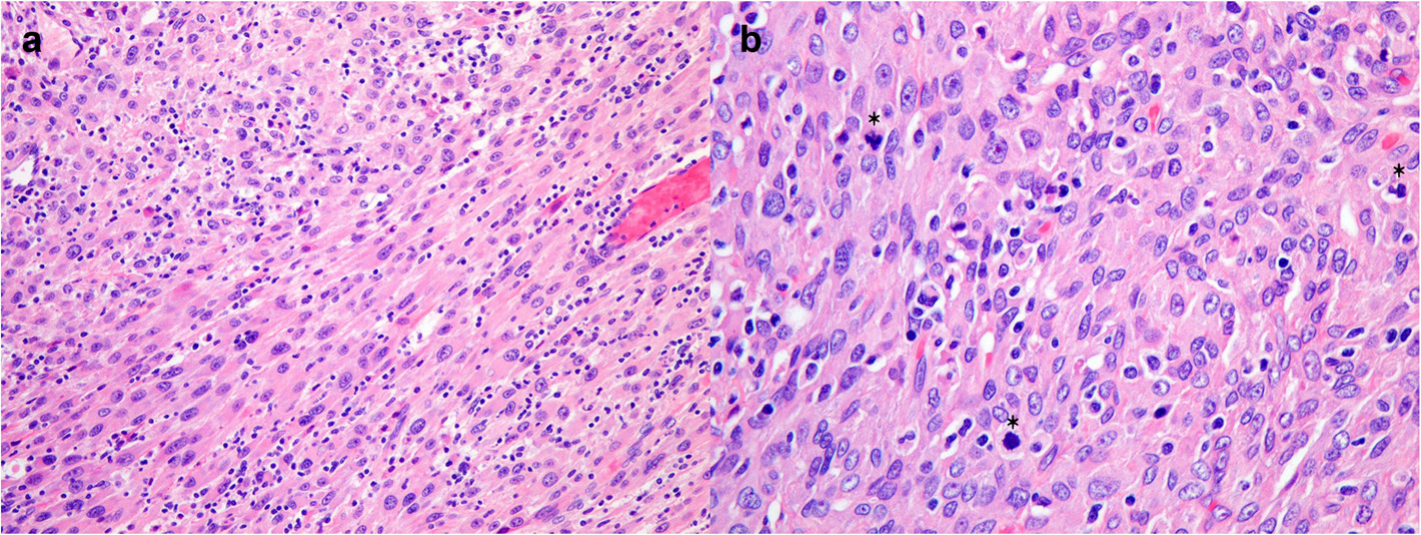
Photomicrograph showing (a) epithelioid spindled cells with abundant eosinophilic cytoplasm in a fascicular pattern and (b) increased mitotic activity (*) with typical and atypical mitoses.

Case 3 in Tables 2 and 3 is a 65-year-old male who, during work-up for claudication, was found incidentally to have a large adrenal mass as well as a renal mass. He had an open radical nephrectomy. The suprarenal tumor measured 12.8 × 8.9 × 7.5 cm. Microscopically, the suprarenal tumor was composed of solid sheets of epithelioid cells with abundant eosinophilic cytoplasm and spindled cells, which in some areas formed a fascicular growth pattern as seen in Figure 1. There was marked nuclear pleomorphism including occasional malignant multinucleated giant cells. There was no heterologous differentiation although a few cells had eosinophilic rhabdoid-like globules. Both typical and atypical mitoses were identified with an average of 20 mitoses per 20 HPF as seen in Figure 1b. Based on WHO recommendations, this tumor with a spindled morphology qualified for being a sarcomatoid variant [7].This patient remains disease-free at 4 months.

**Figure 2:**
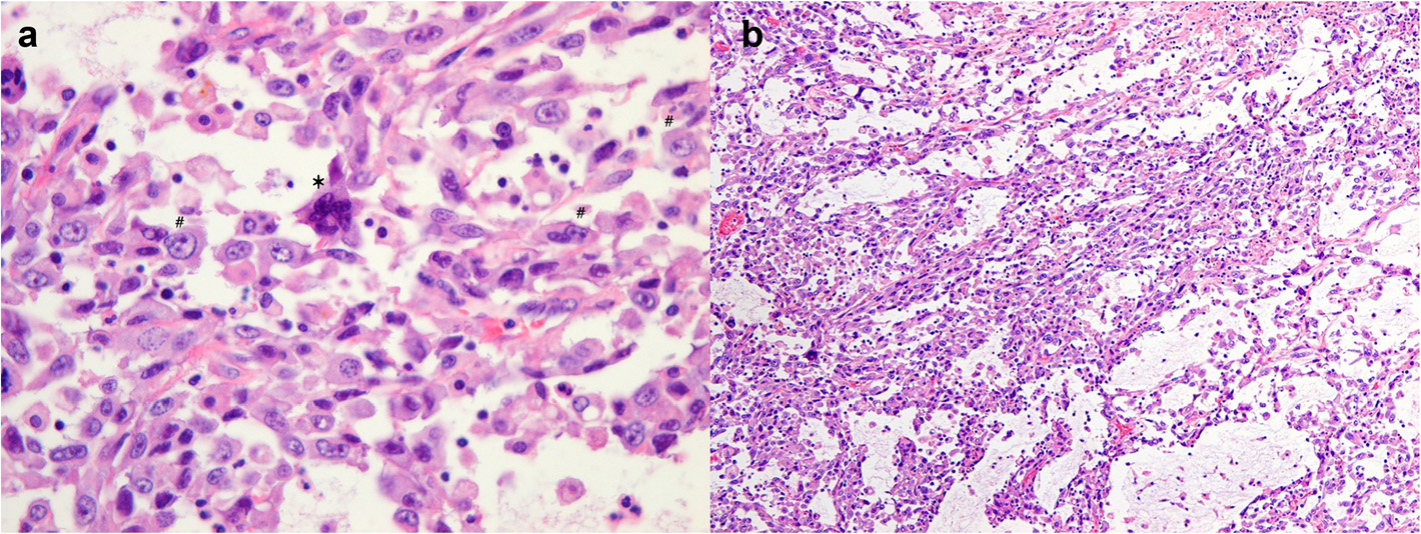
Photomicrograph showing (a) malignant cells with marked nuclear pleomorphism (#) and the presence of a multinucleated tumor giant cell (*) and (b) sarcomatoid areas with spindled cells in a myxomatous background.

Case 7 in Tables 2 and 3 is a 68-year-old lady who was incidentally found to have a large adrenal tumor during follow-up imaging for a previous lung adenocarcinoma. She had a radical nephrectomy. The adrenal tumor measured 13.0 × 11.0 × 9.0 cm. Microscopically, much of the tumor was hemorrhagic and necrotic. The neoplasm showed marked pleomorphism with frequent multinucleated giant cells as seen in Figure 2a. There were epithelioid areas and sarcomatoid areas with spindled cells in a myxomatous background as seen in Figure 2b. There were numerous typical mitoses, five per single HPF in some areas, and atypical mitoses. In agreement with WHO recommendations, this is a sarcomatoid carcinoma [7]. This patient had disease recurrence invading the gastroduodenal artery within 68 days. She required multiple transfusions and embolization of the artery for control of bleeding. She succumbed to her disease 223 days after initial surgery.

## Discussion

### Epidemiology and clinical presentation

Adrenocortical carcinoma is a rare malignancy with an annual incidence of 0.5 to 2.0 per million people with a female-to-male ratio of 1.2 to 1.5:1 [1-4]. The average age of presentation reported by Bilimoria *et al*. (*n* = 3,982) and Kutikov *et al*. (*n* = 4275) in the United States National Cancer Data Base is 55 years [1,4]. This is similar to the average age in our case series of 53.5 years. The demographic information from the five largest published case series (*n* > 300, from unique databases) on ACC, is tabulated in Table 4 [1-4,8-14].

**Table 4:** **Largest ACC case series published to date (** ***n*** **> 300)**

**Reference**	**Published year**	**Country**	**Database**	**Number of patients**	**Average Age (years)**	**Male-to-female ratio**	**Median tumor size (cm)**	**Proportion of tumors with distant metastasis at presentation (%)**
Kutikov *et al*. [4]	2011	United States	National Cancer Database	4,275	54.5	41.9% to 58.1%	11.5	34.4
Kebebew *et al*. [3]	2006	United States	Surveillance, Epidemiology, and End Results (SEER) Database	725	51.2	45.9% to 54.1%	12	34.8
Fassnacht *et al*. [8]	2009	Germany	The German ACC Registry	416	46.7	37.3% to 62.7%	11.3	29.3
Else *et al*. [9]	2014	United States	Michigan Endocrine Oncology Repository	391	47.4	40% to 60%	11.8	29
Kerkhofs *et al*. [2]	2013	Netherlands	Netherlands Cancer Registry	359	56	45% to 55%	Not available	35
Ayala-Ramirez *et al*. [10]	2013	United States	Tumor Registry Database at the University of Texas MD Anderson Cancer Center	330	48.5	35.8% to 64.2%	11 cm	25.8

Cases series with data from identical databases were not included. In this manuscript, we have elected to tabulate publications with the largest *n* from their independent databases. In this context, the following studies that are not represented are as follows: Bilimoria *et al*. (National Cancer Database, *n* = 3,982) [1], Lughezzani *et al*. (SEER, *n* = 573) [11], Sturgeon *et al*. (SEER, *n* = 457) [12], Johanssen *et al*. (German ACC Registry, *n* = 387) [13], and Tran *et al*. (SEER, *n* = 320) [14]. ACC, adrenocortical carcinoma.

Pediatric ACCs have several important differences compared to adult ACCs. The incidence in children is much lower with only 25 new cases of ACC being diagnosed in the USA every year [15]. The incidence for children is greatest in the first year of life [16]. ACC is more common than adrenocortical adenoma in children (approximately 3:1 in newborns and 2:1 in older children) [17,18]. The clinical presentation of these tumors in children is also different with more pediatric patients presenting with symptoms of adrenal hormone hypersecretion. The most common presentation in children is virilization, followed by Cushing’s syndrome [19]. In fetal and newborn patients, the main presentation was an abdominal mass found on physical examination or antenatal sonography, but this is followed closely by virilization [18]. However, pathological diagnosis of pediatric ACC is more challenging because frequently used adult histopathological criteria, such as the Weiss criteria, have not been shown to accurately predict tumor behavior in children and therefore their use is not recommended. Instead, adrenocortical tumors in children should be classified as clinically benign or clinically malignant based on their clinical course [15]. Outcomes in children are better than in adults, with a reported 5-year overall survival of 57%, which is even higher (91.1%) in patients under the age of 5 [16].

Clinical presentation of ACC is variable. While most ACCs are biochemically functional, in many patients this does not manifest clinically, and a large proportion of tumors are discovered incidentally or are metastatic at the time of presentation [20-22], with the most common sites of distant metastasis being, in decreasing frequency, the liver, lungs, and bone [1]. In our series, six out of eight patients presented with incidentally discovered tumors, and only one patient had metastatic disease on presentation. One patient presented with Cushing’s syndrome. The reported proportion of functioning ACC varies in the literature, but the majority is functioning, and most functional tumors secrete cortisol [20-22].

### Diagnosis

#### Imaging

While imaging is not able to definitively diagnose malignancy in an adrenal mass, modern modalities can correctly differentiate adrenal masses before histopathological diagnosis in most cases. The most obvious characteristic noted on cross-sectional imaging of an adrenal mass is the size of the lesion. A cut-off of 4 cm has a sensitivity of 93% for identifying adrenal carcinoma and, while this is a conservative size cut-off, it should be used due to the aggressive nature of ACC and the importance of early diagnosis [5]. Higher cut-offs of 5 or 6 cm have been suggested with sensitivities of 90% or greater in smaller studies [23,24]. Beyond size, few other features of the mass on unenhanced and contrast-enhanced CT help to steer accurate radiographic identification. The density of the adrenal lesion has been proposed as a valuable tool, and benign adenomas tend to be more lipid-rich and have Hounsfield unit densities less than 10 [23,25]. Tumor extension into the IVC with a tumor thrombus is seen in a proportion of tumors, particularly in right-sided tumors, and is indicative of malignancy. On contrast-enhanced CT, little enhancement is seen in the central necrosis of malignant tumors compared to the peripheral tumor [24,25]. Lastly, on contrast-enhanced imaging, the relative percentage of contrast agent enhancement washout seen in malignant tumors after 15 min is generally less than 40% [24-26]. Abdominal CT can be combined with chest imaging in order to establish the presence of any metastatic lung disease. The efficacy of MRI is relatively equivalent to that of CT and in patients where radiation is not a concern, CT scan is recommended as the initial radiographic modality [21,23].

Since not all patients with functioning tumors present with symptoms of hormonal excess, a careful endocrine work-up should be performed and the absence of secretion should alert clinicians to the possibility that the mass is not an ACC [20,21]. In contrast, adrenal adenomas are less likely to be functioning and are generally significantly smaller than adrenal carcinomas when discovered incidentally [5].

Biopsy of adrenal masses has a low diagnostic accuracy and may promote needle track metastases. As such, biopsy is not suggested as part of the diagnostic work-up except in patients with metastatic disease, not scheduled for surgery, in whom the diagnosis remains unestablished or in patients with a suspicious endocrine-inactive adrenal mass and a history of an extra-adrenal malignancy [21]. In patients who do not meet these conditions, biopsy of the adrenal mass unnecessarily delays the diagnosis of malignancy.

### Histopathology

Three histopathological scoring systems for distinguishing benign from malignant ACCs have been proposed. The Hough system employs 12 criteria, 7 histologic and 5 non-histologic. Each criterion is assigned a numeric value, and the total score is predictive of the biologic behavior of the tumor [27]. The Hough criteria are tabulated in Table 5. Slooten *et al*. have also proposed a scoring system [28]. Their system includes seven histological criteria and no non-histological criteria. In contrast to the Hough system, the Slooten criteria are not limited by the availability of clinical findings. The Slooten system also assigns numeric values to its criteria and a total value greater than eight is correlated with tumor behavior [27]. The Slooten criteria are tabulated in Table 6.

**Table 5:** **Hough system criteria**

**Criteria**	**Numeric value**
Histological	
Diffuse growth pattern	0.92
Vascular invasion	0.92
Tumor cell necrosis	0.69
Broad fibrous bands	1.00
Capsular invasion	0.37
Mitotic index (>1/10 HPFs)	0.60
Pleomorphism	0.39
Nonhistologic	
Tumor mass (>100 g)	0.60
Urinary 17-ketosteroids (10 mg/1 g creatinine 24 h)	0.50
Response to ACTH (17-hydroxysteroids increased two times after 50 mcg of IV ACTH)	0.42
Cushing syndrome with virilism, virilism alone, or no clinical manifestations	0.42
Weight loss (>10 lb/3 months)	2.00

ACTH, adrenocorticotropic hormone; HPFs, high-powered fields.

**Table 6:** **van Slooten criteria**

**Criteria**	**Numeric value**
Regressive changes such as necrosis, hemorrhage, fibrosis, or calcification	5.7
Loss of normal structure	1.6
Nuclear atypia	2.1
Nuclear hyperchromasia	2.6
Abnormal nucleoli structure	4.1
Mitotic activity >2/10 HPFs	9.0
Capsular or vascular invasion	3.3

HPFs, high-powered fields.

The Weiss criteria introduced in 1984 [29], later revised [30], and then modified in 2002 [31], are the current standard of practice to establish the diagnosis of ACC. Histopathological diagnosis of ACC is made when tumors meet three of the nine Weiss criteria as listed below [21,30,32]: 1) grade 3 or 4 nuclear grade (enlarged, oval to lobulated nuclei with coarsely granular to hyperchromatic chromatin and easily discernible, prominent nucleoli); 2) mitotic grade >5/50 HPFs; 3) atypical mitoses; 4) clear cells comprising 25% or less of the tumor; 5) diffuse architecture greater than one third of the tumor; 6) necrosis; 7) invasion of venous structures; 8) invasion of sinusoidal structures; and 9) invasion of the tumor capsule.

To simplify the Weiss system, while retaining diagnostic value and improving interobserver reliability, a revised system was proposed by Aubert *et al*. [31]. Their system requires assessment of only five Weiss criteria: i) mitotic grade, ii) percent of clear cells comprising the tumor, iii) abnormal mitoses, iv) necrosis, and v) capsular invasion. Though the total Weiss score has been shown to have high interobserver agreement [31], the interobserver reliability of individual criteria has been criticized [27,33]. In particular, nuclear grade, proportion of clear cells, and architectural assessment may have high variability among different observers. Recently, the reticulin method simplifies the Weiss system. This method requires histochemical staining for reticulin with microscopic examination of the reticulin/basal membrane network. Any tumor with a disrupted reticulin framework as well as the presence of mitosis >5/50 HPFs, necrosis, or venous invasion meets the algorithm’s criteria and is considered malignant [34]. This most recent algorithm has been validated and is shown to have relatively high interobserver agreement [33], albeit lower than reported for the total Weiss score [31]. In light of this finding, we stained specimens in our study for reticulin and seven of the eight tumors had a disrupted reticulin framework. On blinded review, the tumor which had an intact reticulin framework (case 5) also lacked Weiss criteria on examination by the study pathologist. Having been initially reported as having diffuse necrosis, >5 mitotic figures per 50 HPFs, and capsular invasion, these histological features were not seen on re-examination of select slides. Furthermore, the tumor weighed only 46 g (4.7 cm). Therefore, whether this tumor truly represents an adrenal carcinoma remains debatable. Unfortunately, clinical correlation was impossible in this patient because they died of unrelated causes within 1 year of initial diagnosis. An incorrect initial diagnosis is not uncommon in adrenocortical carcinoma. A large audit of cases in Germany identified a high histopathological misclassification rate of 13% [13].

#### Immunohistochemistry

##### TP53

The TP53 tumor suppressor gene, linked with La-Fraumeni syndrome, is frequently mutated in cancers, including adrenocortical carcinoma. In Southern Brazil, the incidence of adrenocortical tumors is unusually high, coinciding with a high prevalence of the germ line TP53 mutation R337H which is present in up to 0.5% of newborns in specific regions of Southern Brazil [35]. Carriers of this particular mutation have been found to have a penetrance of 2.39% to 9.9% for adrenocortical tumors [35,36], with most tumors being carcinomas [35]. Interestingly, this particular TP53 mutation was not found to predispose to extra-adrenal cancers, although this relationship was not studied rigorously [36]. In Caucasian patients with ACC, TP53 germ line mutation analysis has revealed a frequency of 3.9% in adult patients which suggests a specific role for TP53 in ACC tumorigenesis [37]. Loss of heterozygosity of 17p13, on which TP53 is encoded, is studied as a valuable diagnostic marker. In one series, 74% of ACCs, compared with 14% of adenomas, had loss of heterozygosity at 17p13 [38]. Gicquel *et al*. also noted the value of 17p13 loss of heterozygosity as an independent predictor of disease-free survival [39].

Immunohistochemical staining for p53 expression in ACC has found aberrant nuclear staining in a varying proportion of carcinomas, 5% to 60% [40-42]. As such, use of p53 expression to help distinguish benign from malignant adrenal tumors may be an important adjuvant to the current histopathological criteria. This is particularly true since aberrant expression is extremely rare in adenomas [41-43]. Patients with increased p53 staining tend to have higher grade tumors, reflected by higher Ki-67 expression, higher tumor stage, and poorer disease-free survival [44]. However, p53 has not been shown to be related to overall survival [44], and its correlation with poor clinical outcomes is likely due to its association with higher tumor grade. In our series, five of the eight tumors had >10% cells with aberrant expression of p53, and the two cases with highest aberrant p53 expression (>85%) also had the highest proliferative activity (Ki-67 > 80%).

##### SF-1

SF-1 expression is specific to the adrenal cortex [45]. It plays an important role in adrenal development and remains expressed into adulthood [46]. Consequently, adrenal tumorigenesis is influenced by the proliferative effect of SF-1, and SF-1 overexpression has been shown in tumors originating from the adrenal cortex [45,47]. In particular, this has been noted in childhood adrenal tumors where an increased copy of the SF-1 gene is associated with tumorigenesis [48,49].

SF-1 immunohistochemical staining has particular clinical value because its expression has high sensitivity and specificity in determining adrenocortical origin of an adrenal mass [45]. As such, it should be used to differentiate adrenal masses [21]. Interestingly, SF-1 has been found to have independent prognostic value in multivariate analysis. Strong SF-1 expression is associated with poor clinical outcome in ACC even after adjustment for stage [45,47]. In the future, the utility of SF-1 may extend to treatment. Inverse agonists of SF-1 have been studied *in vitro* on human ACC cells with success and may have some clinical utility, although this is yet to be trialed [50].

**Figure 3:**
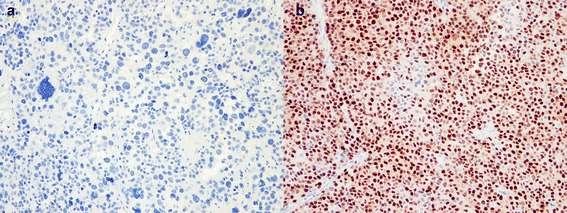
**Photomicrograph of SF-1 stained slide showing (a) adrenocortical carcinoma demonstrating positive staining for SF-1 and (b) sarcomatoid region of adrenocortical carcinoma with absent staining for SF-1.**

Five out of eight of our cases stained positively for SF-1. Interestingly, case 3 and 7, which were histologically sarcomatoid, did not stain for SF-1 in the sarcomatoid areas of the tumors. This is shown in Figure 3a,b. Given the role of SF-1 in adrenocortical cellular development, the lack of SF-1 staining in sarcomatoid adrenal tumors suggests an alternative pathway of development for these tumors. These findings suggest that sarcomatoid adrenocortical carcinoma may arise from a *de novo* rather than a dedifferentiation mechanism. SF-1 staining was unfortunately not performed in other published cases of sarcomatoid ACC. This finding needs to be confirmed in the future, potentially in a meta-analysis of published sarcomatoid ACC cases with SF-1 staining.

##### Β-catenin

Wnt/Β-catenin signaling is thought to be integral to adrenal gland cellular growth and regulation [51]. Activation of this pathway has been shown to be an important factor in tumorigenesis in both benign and malignant tumors of the adrenal cortex [21,51,52]. Β-catenin is normally found on the plasma membrane, associated with E-cadherins, or in the cytoplasm and nucleus where its accumulation influences the Wnt signaling pathway. When accumulation is abnormally increased due to activating mutations, there is abnormal Wnt pathway activation resulting in tumor formation [51]. This has been demonstrated in a mouse model, where constitutive activation of Β-catenin in the adrenal glands has been shown to induce adrenocortical cell hyperproliferation and loss of differentiation [53]. In the older mice, this process resulted in malignant transformation [53].

Immunohistochemical staining for Β-catenin can identify abnormal activation, demonstrated as cytoplasmic or nuclear staining. Both adenomas and carcinomas have been shown to have abnormal Β-catenin staining, with more diffuse and frequent immunostaining in malignant tumors [54]. Abnormal Β-catenin staining has been associated with high-grade ACC, correlating with both high mitotic rate [55] and poor prognosis [56,57]. However, in multi-variate analysis, when the effects of tumor grade are considered, Β-catenin has not been shown to have independent prognostic value [55]. In our series, cytoplasmic staining for Β-catenin was found in 50% of the tumors, including tumors with increased co-expression of Ki-67.

##### Inhibin

Adrenal inhibins have been shown to stain specifically to the adrenal cortex, and particularly to the zona reticularis, with weaker staining in the zona fasciculata and no staining in the zona glomerulosa [58]. Thus, immunohistochemical staining for adrenal inhibin expression is useful to distinguish tumors of adrenal origin. In an early study, Fetsch *et al*. demonstrated immunoreactivity to an adrenal inhibin antibody in all adrenocortical carcinomas and adenomas [59]. However, later studies revealed that a proportion of adrenal tumors are immunonegative, and that negative staining does not exclude the diagnosis of ACC [58,60]. High expression of adrenal inhibin is seen particularly in virilising tumors [58]. Unfortunately, immunopositivity to adrenal inhibin antibodies does not help distinguish benign from malignant adrenal tumors [58,60]. In our series, only three of eight tumors stained positively for adrenal inhibin.

##### IGF

Genetic alterations at the 11p15 chromosomal locus result in an overexpression of IGF-2 in adrenal cortex cells and are fairly specific to both sporadic and syndromic adrenocortical carcinomas but not adenomas [43,52,55,61]. Signaling of IGF-2 through its receptor IGF-1R is thought to play an important role in ACC tumorigenesis [61]; however, recent *in vivo* experiments have demonstrated that isolated over expression of IGF-2, while specific for malignant cells, is not a driving factor in ACC malignant progression [52]. Nonetheless, IGF-2 and IGF-1R antagonists are considered targets for drug development. IGF-1R antagonists have been tested *in vitro* with promising results on human ACC cells [62,63]. Unfortunately, there has been little success with IGF antagonists *in-vivo* [52,64].

#### Pathogenesis

##### Syndromal ACC

ACC, particularly in children, is often associated with familial syndromes [65,66]. La-Fraumeni syndrome, associated with germ line mutations in the TP53 gene, is present in the majority of children with adrenocortical tumors but is seen less commonly in adults [37,65]. Patients with Beckwith-Wiedemann syndrome have overexpression of IGF-2 due to loss of the maternal locus, 11p15 [66]. This mutation is associated with tumors exclusive to childhood and frequently results in benign, and rarely malignant, adrenal tumors [65]. Although the majority of adult ACC has been thought to be sporadic, there is growing evidence of the co-existing presence of hereditary cancer syndromes such as Lynch syndrome [67], multiple endocrine neoplasia type 1 [68], and familial adenomatous polyposis syndrome [56].

#### Staging and prognosis

Identification of tumor features which stratify patients into high and low risk groups is of upmost clinical importance. The most obvious is tumor stage which can be characterized by either the older International Union Against Cancer system or the newer European Network for the Study of Adrenal Tumors (ENS@T) system. The ENS@T system has demonstrated better prognostic stratification and is currently more widely used. It is summarized as follows with corresponding 5-year disease-specific survivals [8,11]:

- Stage I - tumor size less than or equal to 5 cm; 82%.
- Stage II - tumor size greater than 5 cm; 58%.
- Stage III - any tumor size with at least one positive lymph node or tumor infiltrating into surrounding adipose tissue or adjacent organs including the presence of venous tumor thrombus in the inferior vena cava or renal vein; 55%.
- Stage IV - any metastatic disease; 18%.

Further stratifying prognosis beyond disease stage is an important area of ACC research. Weiss first noted the importance of mitotic figures as one of the most useful criteria in distinguishing benign from malignant tumors [29], and Volante *et al*. further suggested stratifying patients into prognostic groups based on mitotic grade with a cutoff of 9 per 50 HPF indicating a high risk tumor [34]. In metastatic ACC, a higher mitotic rate cutoff of 20 per 50 HPF has been shown to be useful in predicting poor outcome [69]. Recently, immunohistochemical analysis of the cellular proliferation marker Ki-67 has been recognized as a more reliable method than the mitotic index in diagnosing ACC and stratifying prognosis [21,70,71]. Few other tumor features have been identified as having possible prognostic importance. One might expect that functioning tumors may have better prognosis due to clinical cues and earlier diagnosis, however, in addition to higher patient age at diagnosis, functional tumors are independently associated with poorer survival [10,20,72]. In metastatic ACC, involvement of fewer tumoral organs is a predictor of better disease-specific survival [69]. As discussed previously, loss of heterozygosity at 17p13 and SF-1 protein staining have been shown to be stage-independent prognostic factors, while p53 and Β-catenin staining have not. As such, there are relatively few useful independent prognostic markers for ACC and a nomogram using only three variables (age, stage, and surgical status) achieved up to 80% accuracy for survival prediction in a large cohort (*n* = 205) of patients [73].

## Conclusions

Despite significant advancement in the past 15 years, accurate diagnosis of ACC remains challenging. The Weiss criteria remain the gold standard for histopathological diagnosis, but lack of interobserver reliability in their assessment has led to the emergence of newer techniques such as the reticulin method. Even in our case series, the challenge of ACC diagnosis is demonstrated. We identified one patient where the initial diagnosis of ACC may have been incorrect on re-examination of histopathological and reticulin staining criteria (case 5). Immunohistochemistry is a growing area of research and several markers have been identified to have clinical value. SF-1 overexpression establishes the adrenal cortex as the origin of adrenal tumors, and the intensity of SF-1 staining is related to prognosis. Interestingly, in our series, sarcomatoid regions in cases 3 and 7 did not express SF-1. This raises the possibility of a *de novo* mechanism of pathogenesis for these rare and highly aggressive tumors. Accuracy in stratification of malignant adrenocortical tumors may be improved by staining for Β-catenin and p53 which are more frequently expressed in ACC but have not been found to have independent prognostic value.

Our case series adds eight cases to the existing literature, with very high expression of p53 and Β-catenin seen in the cases with the highest proliferative activity. Sarcomatoid ACC is extremely rare with very few cases reported in the literature, and these tumors are usually associated with a very poor patient outcome. The published cases, in the English literature, of sarcomatoid ACC are tabulated in Table 7 [7,74-88]. In summary, as adrenocortical carcinoma is a rare disease, we recommend future multicenter studies with appropriate sample sizes to further evaluate and identify reliable diagnostic and prognostic markers.

**Table 7:** **Features of sarcomatoid adrenal cortical carcinomas reported to date**

**Reference**	**Age**	**Sex**	**Clinical presentation**	**Laterality**	**Size**	**Treatment**	**Time to recurrence**	**Time to death**
Okazumi *et al*. [74]	46	M	Abdominal distention + back pain	Right	14 cm	Right adrenalectomy and nephrectomy followed by removal of the tumor thrombus	5 months	206 days
Collina *et al*. [75]	68	F	Abdominal discomfort	Right	11 cm	Surgical resection followed by radiotherapy after tumor recurrence	2 months	6 months
Decorato *et al*. [76]	42	F	Abdominal pain	Left	19 cm	Surgical resection	3 months	7 months
Fischler *et al*. [77]	29	F	Virilization	Left	12.5 cm	Nephroadrenalectomy and splenectomy followed by systemic chemotherapy (cisplatin and etoposide) after recurrence	4 months	8 months
Barksdale *et al*. [78]	79	F	Severe hypertension	Right	9 cm	Right adrenalectomy and cavotomy	4 months	Not reported
Lee *et al*. [79]	61	M	Flank pain + hypertension	Right	12 cm	Radical nephrectomy and right hepatic lobectomy	No recurrence noted	2 days
Sturm *et al*. [7]	31	M	Abdominal pain	Left	12 cm	Adrenalectomy followed by systemic chemotherapy (VP16-cisplatinum) after recurrence	2 months	3 months
Coli *et al*. (2009) [80]	75	F	Abdominal pain	Left	15 cm	Adrenalectomy and splenectomy	3 months	12 months
Feng *et al*. [81]	72	M	Left lumbar pain	Left	7.1 cm on CT scan	Surgical resection	Not reported	Not reported
Sasaki *et al*. [82]	45	M	Abdominal pain, fever, nausea, vomiting, anorexia, hypertension	Left	17 cm	Radical nephrectomy, splenectomy, distal pancreatectomy, left partial colectomy, and wedge biopsy of one hepatic lesion	Hepatic metastasis at presentation. Locoregional recurrence at 3 months	3 months
Bertolini *et al*. [83]	23	F	Incidentally during work-up of metastatic rectal mass	Left	14 cm	Left adrenalectomy with systemic chemotherapy for metastatic rectal cancer	Not reported, however patient had metastatic lesions on presentation which were presumed to be rectal cancer based on the co-existence of a metastatic rectal cancer lesion in the adrenal gland	14 months
Thway *et al*. [84]	45	M	Abdominal bloating + back pain	Left	24 cm	Left radical nephrectomy and splenectomy followed by palliative chemotherapy (vincristine, ifosfamide, carboplatin, doxorubicin, and etoposide)	Metastatic at presentation	11 months
Yan *et al*. [85]	72	M	Flank pain	Right	13 cm	Adrenalectomy	2 years	2.5 years
Kao *et al*. [86]	48	F	Abdominal pain + hypokalemia + weight loss	Right	15 cm	Adrenalectomy, partial nephrectomy, and partial hepatectomy followed by systemic chemotherapy (cisplatin and ifosfamide) after distant metastasis	2 months	Alive with disease at 7 month follow-up
Mark *et al*. [87]	58	M	Flank pain	Right	12 cm	Radial nephrectomy followed by eternal beam radiotherapy to the tumor site	Not reported after 16 month follow-up	Not reported after 16 month follow-up
Shaikh *et al*. [88]	62	F	Abdominal pain	Right	6.5 cm	Adrenalectomy	3 months	4 months

CT, computed tomography; F, female; M, male.
